# Adaptation of fuel selection to acute decrease in voluntary energy expenditure is governed by dietary macronutrient composition in mice

**DOI:** 10.14814/phy2.15044

**Published:** 2021-09-23

**Authors:** Nikhil S. Bhandarkar, Rotem Lahav, Nitzan Maixner, Yulia Haim, G. William Wong, Assaf Rudich, Uri Yoel

**Affiliations:** ^1^ Department of Clinical Biochemistry and Pharmacology Faculty of Health Sciences Ben‐Gurion University of the Negev Beer‐Sheva Israel; ^2^ Department of Physiology Johns Hopkins University Baltimore Maryland USA; ^3^ Soroka University Medical Center Beer‐Sheva Israel

**Keywords:** COVID‐19, energy expenditure, high‐fat diet, metabolic cages, running wheel

## Abstract

In humans, exercise‐induced thermogenesis is a markedly variable component of total energy expenditure, which had been acutely affected worldwide by COVID‐19 pandemic‐related lockdowns. We hypothesized that dietary macronutrient composition may affect metabolic adaptation/fuel selection in response to an acute decrease in voluntary activity. Using mice fed short‐term high‐fat diet (HFD) compared to low‐fat diet (LFD)‐fed mice, we evaluated whole‐body fuel utilization by metabolic cages before and 3 days after omitting a voluntary running wheel in the cage. Short‐term (24–48 h) HFD was sufficient to increase energy intake, fat oxidation, and decrease carbohydrate oxidation. Running wheel omission did not change energy intake, but resulted in a significant 50% decrease in total activity and a ~20% in energy expenditure in the active phase (night‐time), compared to the period with wheel, irrespective of the dietary composition, resulting in significant weight gain. Yet, while in LFD wheel omission significantly decreased active phase fat oxidation, thereby trending to increase respiratory exchange ratio (RER), in HFD it diminished active phase carbohydrate oxidation. In conclusion, acute decrease in voluntary activity resulted in positive energy balance in mice on both diets, and decreased oxidation of the minor energy (macronutrient) fuel source, demonstrating that dietary macronutrient composition determines fuel utilization choices under conditions of acute changes in energetic demand.

## Graphical abstract

Acute decline in voluntary energy expenditure, as occurred in COVID19‐related lockdowns, results in mice in changes in oxidation fuel selection: Low‐fat fed mice decreased fat oxidation during the active phase of the day; Short‐term high‐fat fed mice decreased carbohydrate oxidation.

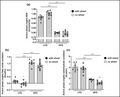

**New & noteworthy**

Acute decrease in voluntary activity resulted in positive energy balance in mice on both low‐ and high‐fat diets (LFD, HFD, respectively). Yet, dietary macronutrient composition determined fuel selection adaptation: mice on LFD decreased fat oxidation during the active phase of the day, while those on HFD reduced carbohydrate oxidation. Thus, acute decrease in voluntary activity decreased oxidation of the minor calorie macronutrient source in either diet.

## 1 INTRODUCTION

Regular physical activity has beneficial effects on multiple aspects of health, and is recommended, by various health organizations, for the general population (Chung, 2020; O'Donovan et al., 2010; Piercy et al., 2018; Singh et al., 2020). Physical activity promotes weight loss, lowers glycated hemoglobin and blood pressure, and improves lipid disorders (Gerstein, 2013; Knowler et al., 2002), effects which are mainly mediated by a reduction in the degree of insulin resistance (Myers et al., 2019). Moreover, when voluntary running wheel freely allowed, it protected C57BL/6J mice from high‐fat diet (HFD)‐induced expression of pro‐inflammatory cytokines, chemokines, and liver macrophage infiltration (Gehrke et al., 2019).

How voluntary energy expenditure (VEE) interacts with dietary energy sources through oxidation (mainly lipid and carbohydrate) is a field of active research (Purdom et al., 2018). In mice, exercise, particularly after feeding, minimized increases in body and adipose tissue weights induced by HFD (Sasaki et al., 2014). In humans increased fat oxidation was induced by aerobic physical activity during short‐term HFD, even a single high‐fat meal (Gregory et al., 2011; Hansen et al., 2007). Longer adaptation to HFD in a 7 weeks study resulted in increased fat oxidation during aerobic exercise in untrained male participants, attributed to utilization of plasma triglycerides (Helge et al., 2001). Taken together, HFD increases reliance on fat oxidation, and decreases carbohydrate oxidation during submaximal exercise, demonstrating a close link between dietary composition and skeletal muscle fuel selection during exercise (Howard & Margolis, 2020).

The recurrent lockdowns that characterize the current COVID‐19 pandemic greatly impacted lifestyle patterns, with many reporting negative effects on eating and physical activity, resulting in weight gain (Ferrante et al., 2020; Robinson et al., 2021; Zachary et al., 2020). Interestingly, in contrast to the well‐studied beneficial effects of initiating physical activity on fuel selection and weight regulation, much less is known about the metabolic response to acute reduction in VEE, as occurs during lockdowns, and how it interacts with dietary composition. Using mice and metabolic cages, we assessed whole‐body fuel utilization and fuel oxidation selection before and after omitting a voluntary running wheel. We hypothesized that dietary energy source may shape the metabolic adaptation to acute decrease in VEE.

## 2 MATERIALS AND METHODS

### 2.1 Animals

**FIGURE 1:**
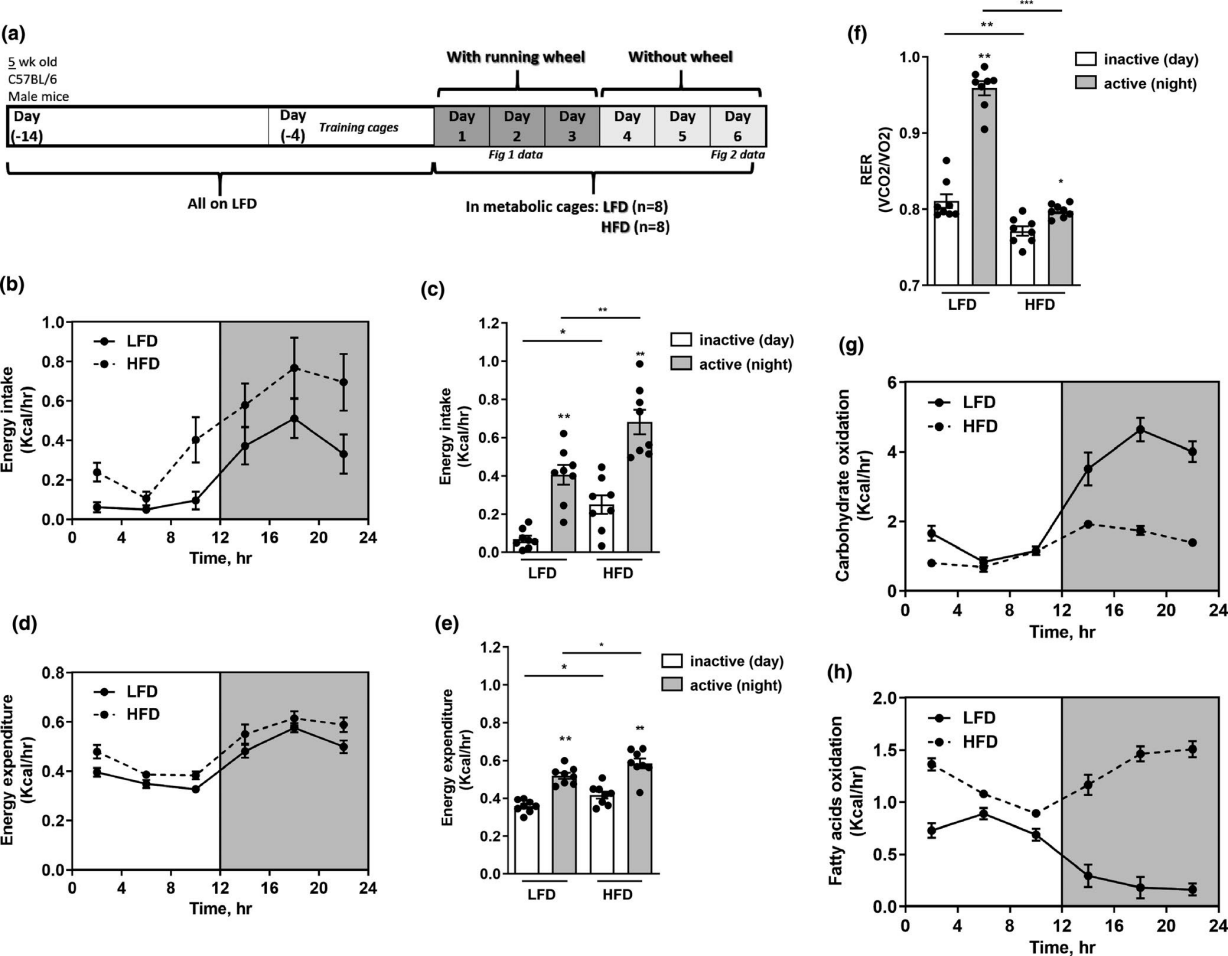
Acute HFD induces a shift in whole‐body fuel utilization. (a) Scheme of study protocol. (b,c) Energy intake and (d,e) Energy expenditure of LFD and HFD mice during active (night) and inactive (day) phases. (b and d) Are hourly averages of energy intake/energy expenditure in each 4‐h interval, and (c and d) are hourly averages of energy intake/energy expenditure per 12 h of active/inactive phases. (f) Hourly average RER of LFD and HFD mice during 12 h of active/inactive phases. Hourly averages of carbohydrate (g) and fatty acid (h) oxidation every 4 h in LFD and HFD mice. Data represent the mean ± SEM from eight mice per group. */**p < 0.05/0.01 within the dietary groups, bar + */**p < 0.05/0.01 between the dietary groups

The study was approved in advance by Ben‐Gurion University Institutional Animal Care and Use Committee (IL‐28‐06‐2017), and was conducted according to the Israeli Animal Welfare Act following the guidelines of the Guide for Care and Use of Laboratory Animals (National Research Council 1996). Five‐week‐old male C57BL/6J mice (Envigo Laboratories, Rehovot, Israel) were housed, 2 mice/cage in a room with constant temperature (23 ± 3°C) and relative humidity, water and food ad‐libitum, and with a 12/12 h light/dark cycle (inactive/active phase, respectively). Mice were fed low‐fat diet (LFD) containing 10% energy from fat (D12450J, Research Diets, Inc., New Brunswick, USA) for a 2 week period, after which they were transferred to Promethion High‐Definition Behavioral Phenotyping System (Sable Instruments, Inc., Las Vegas, NV, USA). Mice were divided randomly into two groups: a control group that continued to consume LFD, and a group fed with HFD containing 60% energy from fat (D12492, Research Diets, Inc.). The two diets’ composition was identical, apart for corn starch (506 and 0 g/Kg) and Lard (20 and 245 g/Kg) for LFD and HFD, respectively. Twenty‐four hours acclimation period with a free access to food and water and standard 12/12 h light/dark cycle were followed by 72 h sampling duration, after which free‐running wheel device was omitted from the cages, and metabolic recording continued for an additional 72 h (Figure 1a). Data acquisition and instrument control were performed using MetaScreen software version 2.2.18.0, and the obtained raw data were processed using ExpeData version 1.8.6. Measurement of respiratory gases (3 min interval) was performed by the GA‐3 gas analyzer (Sable Systems, Inc., Las Vegas, NV, USA) using a pull‐mode, negative‐pressure system. FR‐8 (Sable Systems, Inc.) controlled and measured the air flow with a rate of 2000 mL/min. Animal positions as well as ambulatory and voluntary activities were monitored simultaneously by calorimetry data collection using the XYZ beam arrays with a beam spacing of 0.25 cm and with or without running wheel in the cage.

### 2.2 Metabolic calculations

RER (respiratory exchange rate), total carbohydrate, and fat oxidation (g/h) were computed from V_CO2_ (L/h) to V_O2_ (L/h) using stoichiometric equations, with the assumption that protein oxidation during exercise was negligible, as follows: Respiratory exchange rate (RER) = V_CO2_/V_O2_ (O'Hara et al., 2017); Carbohydrate oxidation g/h = 4.585 × V_CO2_ − 3.23 × V_O2_; Fat oxidation g/h = 1.69 × V_O2_ − 1.69 × V_CO2_ (O'Hara et al., 2017). Energy intake was calculated, in Kcal/h, as Food intake × calories per gram. Energy expenditure (EE) was calculated as V_O2_ × (3.815 + 1.232 × RER). Body weight was measured before introducing the running wheel, when taking it out of the metabolic cages, and at the end of the study. Given the short duration of the intervention and small differences in body weight between the groups, measurements were not adjusted to body weight.

### 2.3 Statistical analysis

Analyses were performed by using GraphPad Prism 9.1.0. Statistical significance was examined using: non‐parametric unpaired Mann–Whitney *U* test between mice's groups LFD versus HFD, and non‐parametric paired Wilcoxon test between groups with wheel versus without wheel; and active phase (night) versus inactive phase (day). A *p* ≤ 0.05 was considered to be a statistically significant difference. All data shown are mean ± SEM.

## 3 RESULTS

### 3.1 Rapid metabolic adaptation to HFD

Seven‐week‐old C57BL/6J mice were placed individually in metabolic cages after a 2‐week adaptation to LFD and 4 days adaptation to metabolic cages, and then fed either LFD or HFD (Figure 1a). The initial 3 days of dietary intervention were in the presence of a free‐running wheel, to allow mice to run ad‐libitum, after which the wheel was taken out of the cage, and metabolic cage recordings continued for an additional 3 d period (Figure 1a).

Mice on either diet exhibited a significant diurnal (day‐night/inactive‐active phase, respectively) difference in Energy Intake (EI, Figure 1b,c), Energy Expenditure (EE, Figure 1d,e), and in the respiratory exchange ratio (RER, Figure 1f). Yet, mice switched to HFD, compared to mice continuously fed LFD, displayed a significant increase in EI and in EE, in both the active and inactive phases of the day. Interestingly, the magnitude of this increment was grater in EI compared with EE in both day phases. In addition, within 24–48 h of initiating HFD (i.e., day 2), a significant shift in fuel utilization was observed: respiratory exchange rate (RER) significantly declined by HFD during both active and non‐active phases (Figure 1f), and the inactive‐active phase difference significantly diminished compared to LFD mice. This is consistent with lower metabolic flexibility and a greater relative reliance on fat oxidation throughout the day. Indeed, short‐term HFD‐induced marked change in calculated carbohydrate and fat oxidation rates (Figure 1g,h, respectively): Mice on LFD relied more on fat oxidation during inactive (day, sleep) phase and on carbohydrate oxidation during the active (night, feeding) phase. In contrast, HFD induced a marked reliance on fat oxidation for energy production, and the inactive‐active difference in fuel selection was markedly attenuated compared to mice on LFD.

### 3.2 Running wheel omission equally diminishes active phase (night‐time) activity in LFD and HFD mice

**FIGURE 2:**
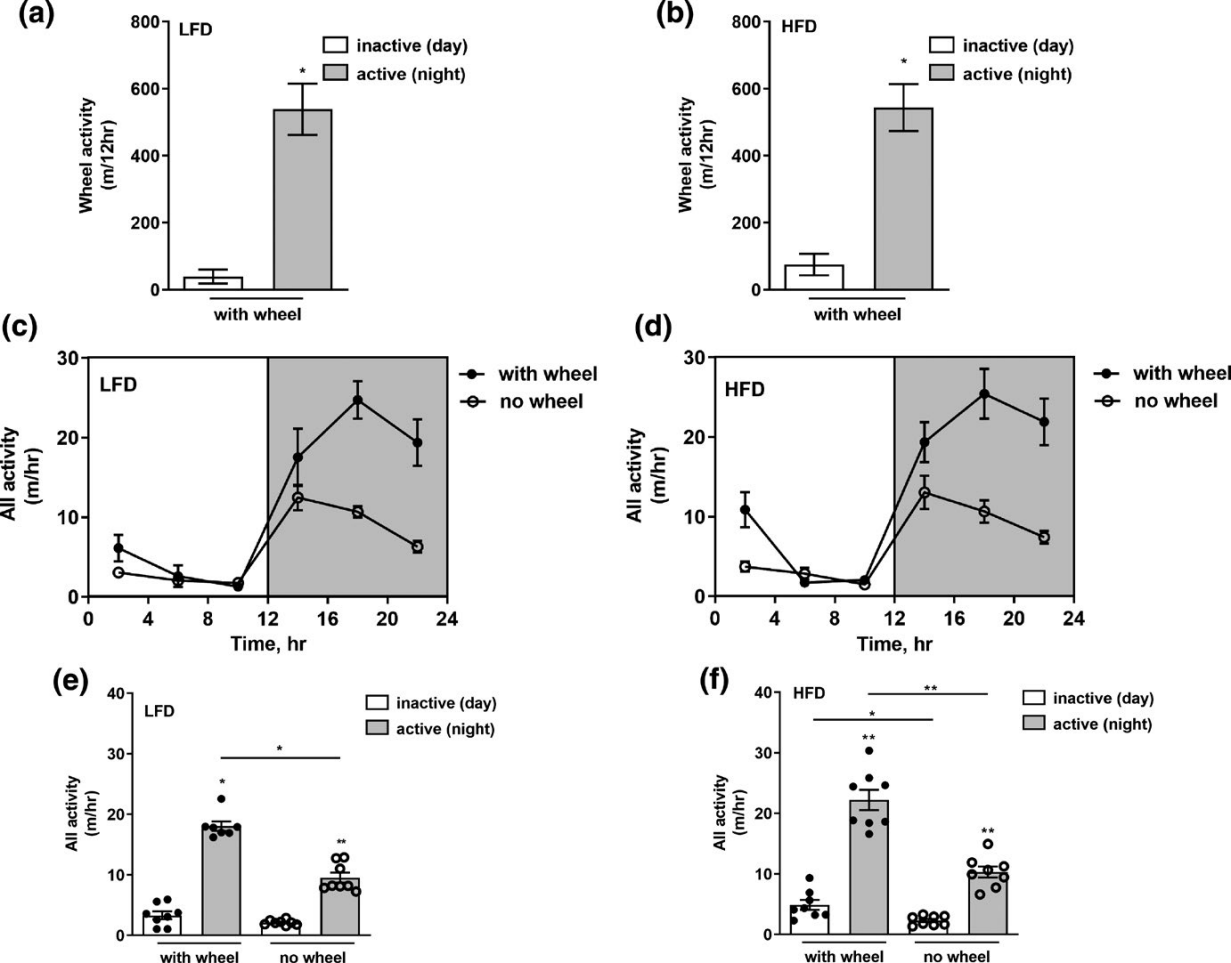
Effect of running wheel omission on voluntary and total activity. Cumulative wheel activity in active (gray bar) and inactive (white bar) phases in LFD (a) and HFD (b) mice. (c,d) Mean hourly activity every 4 h period in LFD and HFD mice, respectively, before and after running wheel omission from the cage. (e,f) Mean hourly activity per 12 h day phase in LFD and HFD mice, respectively, with (full black dots) or without (empty white dots) presence of the wheel. Data represent the mean ± SEM from 4–8 mice per group at each time point. */**p < 0.05/0.01 within the dietary groups, bar + */**p < 0.05/0.01 between the dietary groups

During the first 3d on either LFD or HFD, the metabolic cages included a free‐running wheel. Mice on either diet mainly utilized the wheel during the active phase of the day (night), increasing the wheel running distance by >fivefold compared to the day/inactive phase, reaching ~550 m/12 h (Figure 2a,b). Omitting the wheel from the metabolic cages in subsequent 3 d of measurement similarly affected mice on both diets, as both LFD (Figure 2c,e) and HFD (Figure 2d,f) mice decreased total activity during the active phase (night) by ~50%.

### 3.3 Effect of dietary composition on the metabolic adaptation to acute decrease in voluntary activity

**FIGURE 3:**
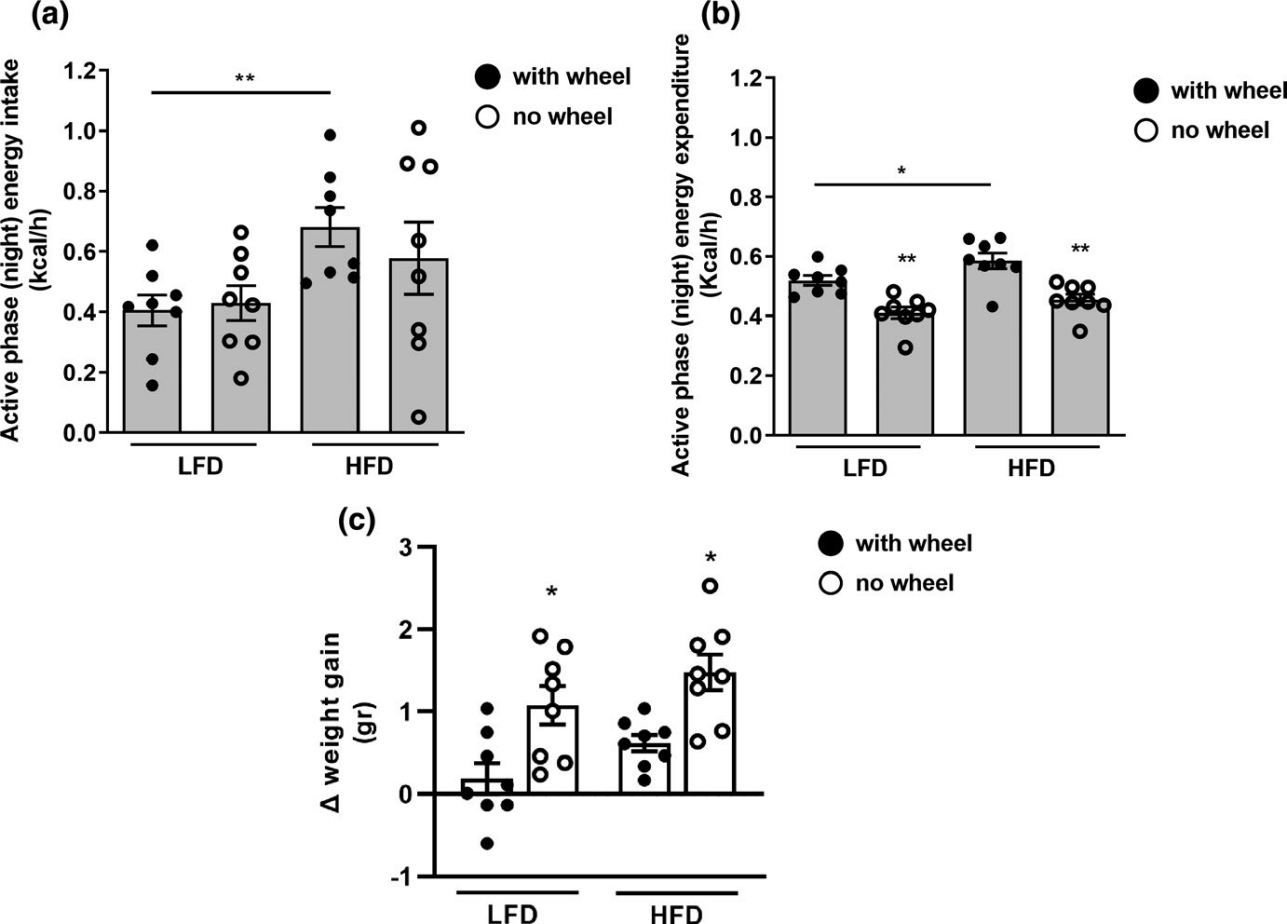
Effect of running wheel omission on energy intake and energy expenditure during the active day phase. Hourly average of energy intake (a) and energy expenditure (b) in the active phase (night) in LFD and HFD mice with (black circles) or without (white circles) the presence of running‐ wheel. (c) Three‐day change in body weight with (black circles) or without (white circles) the presence of running wheel. Data represent the mean ± SEM from eight mice per group. */**p < 0.05/0.01 within the dietary groups, bar + */**p < 0.05/0.01 between the dietary groups

We next determined how the acute decline in active phase (night‐time) voluntary activity induced by running wheel omission affected energy intake, expenditure, and fuel utilization in mice on either LFD or HFD. The trend for increased EI in HFD compared to LFD mice (Figure 1b,c) was still evident in the active phase regardless of the presence of the wheel (Figure 3a). Interestingly, mice on either diet did not, on average, acutely decrease their active phase EI upon wheel omission, despite the acute decline in total activity. Yet, EE during the active phase significantly declined after wheel omission compared to EE when running wheel was present, regardless of the diet (Figure 3b). There was a comparable positive energy balance in both groups caused by wheel omission, with active phase energy balance increasing by 1.15 and 0.76 Kcal/12 h for LFD and HFD mice, respectively, *p* = 0.819. Correspondingly, wheel omission significantly increased 3 d weight gain in both LFD and HFD mice compared to weight gain in the preceding, 3 d with wheel, period (Figure 3c). Thus, acute decline in voluntary activity during the active phase decreased active phase EE but not EI resulting in positive energy balance and increased weight gain during the active phase of the day, irrespective of dietary macronutrient composition.

**FIGURE 4:**
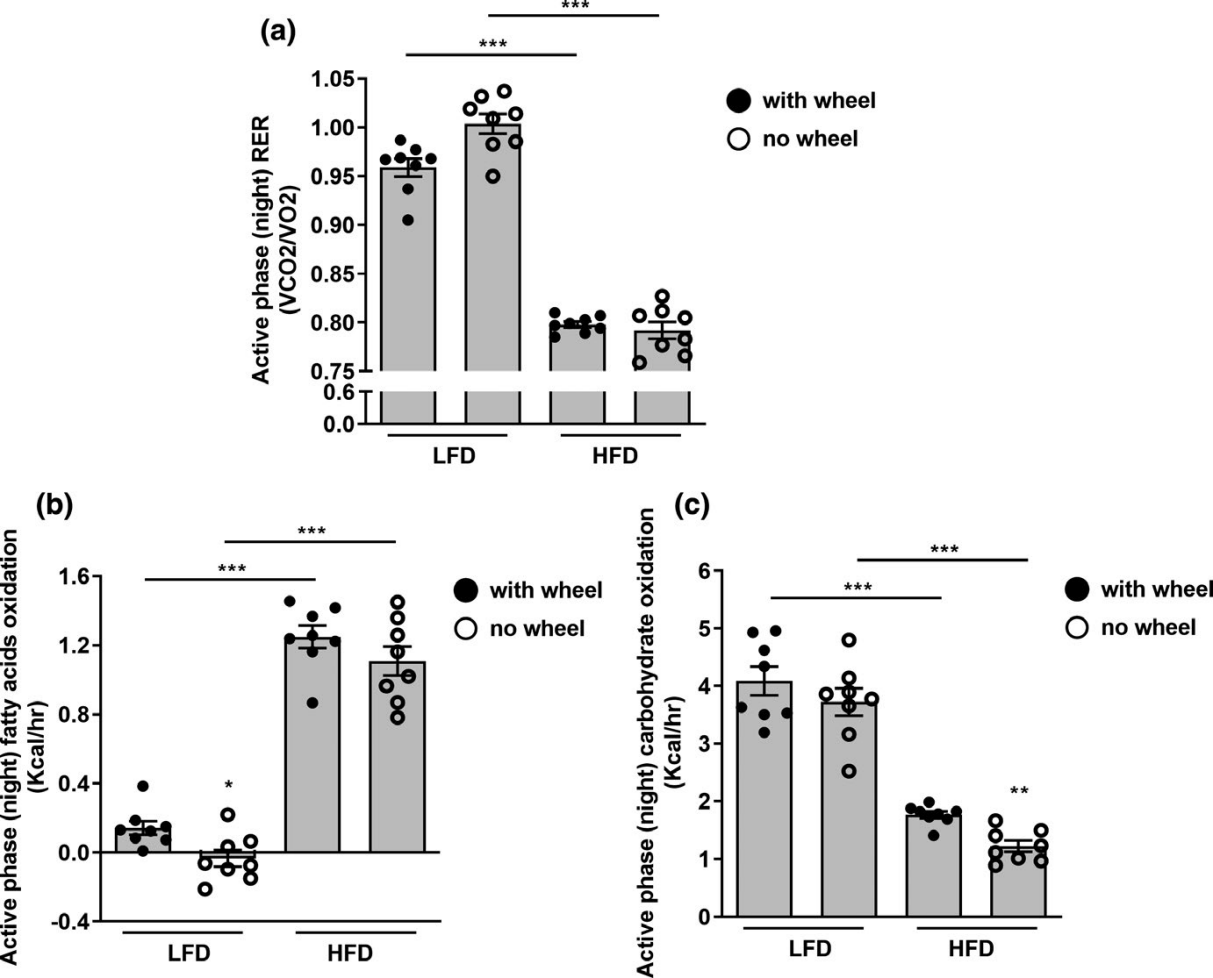
Effect of acute decrease in voluntary activity on fuel utilization in LFD and HFD mice. Hourly average RER (a), fatty acid (b), and carbohydrate (c) oxidation during active phase (night) in LFD and HFD mice with (black circles) or without (white circles) the presence of running wheel. Data represent the mean ± SEM from eight mice per group. */**p < 0.05/0.01 within the dietary groups, bar + */**p < 0.05/0.01 between the dietary groups

We next evaluated how an acute decline in voluntary activity during the active phase of the day, as modeled by wheel omission, affected fuel utilization. Active phase RER trended to increase in mice on LFD, and exhibited a minor decline in mice on HFD (Figure 4a). Interestingly, this trend to increase RER in the LFD mice in response to wheel omission could be attributed to a significantly decreased fat oxidation during the active phase of the day (Figure 4b), without significantly affecting carbohydrate oxidation (Figure 4c). In HFD mice an acute decrease in voluntary activity during the active phase of the day diminished significantly carbohydrate oxidation (Figure 4c), with no significant effect on fat oxidation (Figure 4b). Thus, in mice on either diet, utilization of the minor dietary fuel source was diminished by acute decrease in voluntary activity during the active phase.

## 4 DISCUSSION

In the current study we evaluated changes in fuel oxidation selection when omitting free‐running wheel in mice fed either LFD or HFD. When running wheel was freely used, mice on HFD for >24 h significantly increased both EI and EE in the active (night) and inactive (day) phases, and switched to energy utilization predominated by fat oxidation. Following running wheel omission, mice on either LFD or HFD similarly decreased total activity during the active phase by ~50%, but did not acutely decrease their active phase EI, resulting in positive energy balance and significantly increased weight gain. Change in fuel utilization depended on dietary composition: wheel omission in LFD mice significantly decreased active phase fat oxidation, while it diminished active phase carbohydrate oxidation in HFD. Thus, dietary composition determined fuel selection, with acute decrease in VEE reducing oxidation of the minor dietary energy source.

Consistent with our results, previous studies demonstrated that short and longer‐term HFD increases both EI and EE, with augmented fat oxidation and consequently reduced RER (Brown et al., 1985; Burke et al., 2021; Che et al., 2021; Gregory et al., 2011; Hansen et al., 2007; Helge et al., 2001; Sasaki et al., 2014). This is driven by increased utilization of intramuscular and plasma triglyceride/non‐esterified fatty acids stores (Helge et al., 2001; Howard & Margolis, 2020). These observations were confirmed in mouse models (Brown et al., 1985; Sasaki et al., 2014) as well as in human studies (Burke et al., 2021; Che et al., 2021; Gregory et al., 2011; Hansen et al., 2007; Helge et al., 2001). Yet, the wheel‐omission‐mediated fuel oxidation changes are beyond merely reflecting the diet, since both diets, though to different proportions, include both carbohydrates and fat, and it is the wheel omission that decreased fuel oxidation specifically of the minor nutritional source, which had been utilized under conditions of greater energy demand. In support of the effect of dietary composition on fuel selection for oxidation, a recent report demonstrated that supplementing moderately HFD with monosaccharides (fructose and glucose), resulted in higher mean RER during the active phase (Bouwman et al., 2020).

In contrast to the established documentation of macronutrient utilization during exercise, we did not find reports evaluating the metabolic effects of acute reduction in VEE. Our finding that on either diet wheel omission resulted in positive energy balance and weight gain may model acute reduction in physical activity, as occurred worldwide during recurrent lockdowns in the COVID‐19 pandemic. Indeed, studies that evaluated lifestyle changes during lockdowns demonstrated negative effects on eating and physical activity, resulting in weight gain in the general population (Ferrante et al., 2020; Micheletti Cremasco et al., 2021; Robinson et al., 2021; Zachary et al., 2020), and specifically in youths (Jia et al., 2021) and adolescents (Androutsos et al., 2021). These detrimental effects reinforce the value of interventions like online structured physical activity programs to restore VEE (Constantini et al., 2021).

Our study has several limitations. First, we evaluated the mice following wheel omission for 3 d only. Thus, we cannot exclude the possibility that with longer time EI would have declined, making energy balance less positive or even neutral. However, a recent survey reporting that reduced physical activity is a risk factor for weight gain during self‐quarantine (Zachary et al., 2020) may serve as an indirect support to our findings, as it suggests that a longer period of reduction in VEE does not significantly reduce the positive energy balance. Moreover, we did not evaluate switching HFD‐fed‐mice back to LFD, or re‐introducing the running wheel. Possibly, switching HFD‐fed‐mice back to LFD may result in gradual change to the oxidation pattern as seen in LFD‐fed‐mice, and re‐introducing the running wheel may increase EE, moderating or reversing the positive energy balance. Fat and carbohydrate oxidation were not adjusted to effective body weight given the short duration of the intervention and modest weight differences between the groups. Furthermore, wheel omission decreased fat or carbohydrate oxidation, depending on the diet, despite positive energy balance and weight gain in both groups, rendering minor changes in effective body weight an unlikely explanation. We did not perform measurements of energy excretion (fecal caloric content). Thus, we cannot rule‐out the possibility that excretion of macronutrients could be acutely altered and contributed to changes in the overall energy balance. In this study we did not assess biochemical parameters such as plasma triglycerides, high‐density lipoprotein, or HOMA‐IR. It would be interesting to follow such parameters as part of a longer‐term study. Finally, the extrapolation of our results into the lifestyle changes seen during lockdowns in the COVID‐19 pandemic in humans should obviously be taken with caution: Additional factors that may directly or indirectly affect energy balance were reported during lockdowns, including inadequate sleep, increased consumption of sugar‐sweetened beverages, alcoholic beverages, increased consumption of snacks, “emotional” eating, and increased social isolation (Al‐Musharaf, 2020; Ferrante et al., 2020; Robinson et al., 2021; Sánchez et al., 2021; Zachary et al., 2020).

In conclusion, in the presence of a running wheel, mice whose diet was changed from LFD to HFD increased both EI and EE. Acute decrease in voluntary activity resulted in mice on either LFD or HFD in a comparable decrease in total activity during the active phase by ~50%, but did not acutely decrease their active phase EI, resulting in positive energy balance and weight gain. In addition, wheel omission decreased oxidation of the minor energy (macronutrient) fuel source, demonstrating that dietary macronutrient composition determines fuel selection under conditions of acute changes in energetic demand.

## CONFLICT OF INTEREST

There is no conflict of interest to declare for any of the co‐authors.

## AUTHOR CONTRIBUTIONS

NSB: Study design, operation of metabolic cages, analysis and interpretation of data, critical review of the manuscript, and approval of the manuscript final version; RL: Operation of metabolic cages, analysis and interpretation of data, critical review of the manuscript, and approval of the manuscript final version; NM: Study design, analysis and interpretation of data, critical review of the manuscript, and approval of the manuscript final version. YH: Study design, operation of metabolic cages, analysis and interpretation of data, critical review of the manuscript, manuscript writing, and final approval. GWW: analysis and interpretation of data, critical review of the manuscript, and approval of the manuscript final version. AR: Study design, analysis and interpretation of data, critical review of the manuscript, manuscript writing and final approval, and correspondence. UY: Analysis and interpretation of data, critical review of the manuscript, manuscript writing and final approval, and correspondence.

## References

[phy215044-bib-0001] Al‐Musharaf, S. (2020). Prevalence and predictors of emotional eating among healthy young Saudi women during the COVID‐19 pandemic. Nutrients, 12, 2923. 10.3390/nu12102923 PMC7598723 32987773

[phy215044-bib-0002] Androutsos, O., Perperidi, M., Georgiou, C., & Chouliaras, G. (2021). Lifestyle changes and determinants of children's and adolescents’ body weight increase during the first COVID‐19 lockdown in Greece: The COV‐EAT study. Nutrients, 13, 930. 10.3390/nu13030930 33805678 PMC7998995

[phy215044-bib-0003] Bouwman, L. M. S., Nieuwenhuizen, A. G., Swarts, H. J. M., Piga, R., van Schothorst, E. M., & Keijer, J. (2020). Metabolic effects of the dietary monosaccharides fructose, fructose‐glucose, or glucose in mice fed a starch‐containing moderate high‐fat diet. Physiological Reports, 8, e14350. 10.14814/phy2.14350 32026655 PMC7002529

[phy215044-bib-0004] Brown, J. D., Naples, S. P., & Booth, F. W. (1985). Effects of voluntary running on oxygen consumption, RQ, and energy expenditure during primary prevention of diet‐induced obesity in C57BL/6N mice. Journal of Applied Physiology, 113(473–478), 2012. 10.1152/japplphysiol.00668.2011 22653990

[phy215044-bib-0005] Burke, L. M., Whitfield, J., Heikura, I. A., Ross, M. L. R., Tee, N., Forbes, S. F., Hall, R., McKay, A. K. A., Wallett, A. M., & Sharma, A. P. (2021). Adaptation to a low carbohydrate high fat diet is rapid but impairs endurance exercise metabolism and performance despite enhanced glycogen availability. Journal of Physiology, 599, 771–790. 10.1113/JP280221 PMC7891450 32697366

[phy215044-bib-0006] Che, K., Qiu, J., Yi, L., Zou, M., Li, Z., Carr, A., Snipe, R. M. J., & Benardot, D. (2021). Effects of a short‐term “fat adaptation with carbohydrate restoration” diet on metabolic responses and exercise performance in well‐trained runners. Nutrients, 13, 1033. 10.3390/nu13031033 33806822 PMC8005046

[phy215044-bib-0007] Chung, E. H. (2020). Editoral commentary: Move more, sit less: Updated guidelines promote any physical activity for all. Trends in Cardiovascular Medicine, 2020(30), 413–414. 10.1016/j.tcm.2019.10.008 31706788

[phy215044-bib-0008] Constantini, K., Markus, I., Epel, N., Jakobovich, R., Gepner, Y., & Lev‐Ari, S. (2021). Continued participation of Israeli adolescents in online sports programs during the COVID‐19 pandemic is associated with higher resilience. International Journal of Environmental Research and Public Health, 18, 4386. 10.3390/ijerph18084386 33924245 PMC8074771

[phy215044-bib-0009] Ferrante, G., Camussi, E., Piccinelli, C., Senore, C., Armaroli, P., Ortale, A., Garena, F., & Giordano, L. (2020). Did social isolation during the SARS‐CoV‐2 epidemic have an impact on the lifestyles of citizens? Epidemiologia E Prevenzione, 44(Suppl 2), 353–362. 10.19191/EP20.5-6.S2.137 33412829

[phy215044-bib-0010] Gehrke, N., Biedenbach, J., Huber, Y., Straub, B. K., Galle, P. R., Simon, P., & Schattenberg, J. M. (2019). Voluntary exercise in mice fed an obesogenic diet alters the hepatic immune phenotype and improves metabolic parameters—An animal model of life style intervention in NAFLD. Scientific Reports, 9, 4007. 10.1038/s41598-018-38321-9 30850619 PMC6408519

[phy215044-bib-0011] Gerstein, H. C. (2013). Do lifestyle changes reduce serious outcomes in diabetes? New England Journal of Medicine, 369, 189–190. 10.1056/NEJMe1306987 23796132

[phy215044-bib-0012] Gregory, S., Wood, R., Matthews, T., Vanlangen, D., Sawyer, J., & Headley, S. (2011). Substrate utilization is influenced by acute dietary carbohydrate intake in active, healthy females. Journal of Sports Science and Medicine, 10, 59–65. 24149296 PMC3737902

[phy215044-bib-0013] Hansen, K. C., Zhang, Z., Gomez, T., Adams, A. K., & Schoeller, D. A. (2007). Exercise increases the proportion of fat utilization during short‐term consumption of a high‐fat diet. American Journal of Clinical Nutrition, 85, 109–116. 10.1093/ajcn/85.1.109 17209185

[phy215044-bib-0014] Helge, J. W., Watt, P. W., Richter, E. A., Rennie, M. J., & Kiens, B. (2001). Fat utilization during exercise: Adaptation to a fat‐rich diet increases utilization of plasma fatty acids and very low density lipoprotein‐triacylglycerol in humans. Journal of Physiology, 537, 1009–1020. 10.1111/j.1469-7793.2001.01009.x PMC2279002 11744773

[phy215044-bib-0015] Howard, E. E., & Margolis, L. M. (2020). Intramuscular mechanisms mediating adaptation to low‐carbohydrate, high‐fat diets during exercise training. Nutrients, 12, 2496. 10.3390/nu12092496 PMC7551624 32824957

[phy215044-bib-0016] Jia, P., Liu, L., Xie, X., Yuan, C., Chen, H., Guo, B., Zhou, J., & Yang, S. (2021). Changes in dietary patterns among youths in China during COVID‐19 epidemic: The COVID‐19 impact on lifestyle change survey (COINLICS). Appetite, 158, 105015. 10.1016/j.appet.2020.105015 33121998

[phy215044-bib-0017] Knowler, W. C., Barrett‐Connor, E., Fowler, S. E., Hamman, R. F., Lachin, J. M., Walker, E. A., Nathan, D. M., & Diabetes Prevention Program Research Group . (2002). Reduction in the incidence of type 2 diabetes with lifestyle intervention or metformin. New England Journal of Medicine, 346, 393–403. 10.1056/NEJMoa012512 PMC1370926 11832527

[phy215044-bib-0018] Micheletti Cremasco, M., Mulasso, A., Moroni, A., Testa, A., Degan, R., Rainoldi, A., & Rabaglietti, E. (2021). Relation among perceived weight change, sedentary activities and sleep quality during COVID‐19 lockdown: A study in an academic community in Northern Italy. International Journal of Environmental Research and Public Health, 18, 2943. 10.3390/ijerph18062943 33805640 PMC8001929

[phy215044-bib-0019] Myers, J., Kokkinos, P., & Nyelin, E. (2019). Physical activity, cardiorespiratory fitness, and the metabolic syndrome. Nutrients, 11, 1652. 10.3390/nu11071652 PMC6683051 31331009

[phy215044-bib-0020] O'Donovan, G., Blazevich, A. J., Boreham, C., Cooper, A. R., Crank, H., Ekelund, U., Fox, K. R., Gately, P., Giles‐Corti, B., Gill, J. M., Hamer, M., McDermott, I., Murphy, M., Mutrie, N., Reilly, J. J., Saxton, J. M., & Stamatakis, E. (2010). The ABC of physical activity for health: A consensus statement from the British association of sport and exercise sciences. Journal of Sports Sciences, 28, 573–591. 10.1080/02640411003671212 20401789

[phy215044-bib-0021] O'Hara, J. P., Woods, D. R., Mellor, A., Boos, C., Gallagher, L., Tsakirides, C., Arjomandkhah, N. C., Holdsworth, D. A., Cooke, C. B., Morrison, D. J., Preston, T., & King, R. F. (2017). A comparison of substrate oxidation during prolonged exercise in men at terrestrial altitude and normobaric normoxia following the coingestion of 13C glucose and 13C fructose. Physiological Reports, 5, e13101. 10.14814/phy2.13101 28082428 PMC5256160

[phy215044-bib-0022] Piercy, K. L., Troiano, R. P., Ballard, R. M., Carlson, S. A., Fulton, J. E., Galuska, D. A., George, S. M., & Olson, R. D. (2018). The physical activity guidelines for Americans. JAMA, 320, 2020–2028. 10.1001/jama.2018.14854 30418471 PMC9582631

[phy215044-bib-0023] Purdom, T., Kravitz, L., Dokladny, K., & Mermier, C. (2018). Understanding the factors that effect maximal fat oxidation. Journal of the International Society of Sports Nutrition, 15, 3. 10.1186/s12970-018-0207-1 29344008 PMC5766985

[phy215044-bib-0024] Robinson, E., Boyland, E., Chisholm, A., Harrold, J., Maloney, N. G., Marty, L., Mead, B. R., Noonan, R., & Hardman, C. A. (2021). Obesity, eating behavior and physical activity during COVID‐19 lockdown: A study of UK adults. Appetite, 156, 104853. 10.1016/j.appet.2020.104853 33038479 PMC7540284

[phy215044-bib-0025] Sánchez, E., Lecube, A., Bellido, D., Monereo, S., Malagón, M. M., Tinahones, F. J., & On Behalf of the Spanish Society for the Study of Obesity . (2021). Leading factors for weight gain during COVID‐19 lockdown in a Spanish population: A cross‐sectional study. Nutrients, 13, 894. 10.3390/nu13030894 33801989 PMC8000852

[phy215044-bib-0026] Sasaki, H., Ohtsu, T., Ikeda, Y., Tsubosaka, M., & Shibata, S. (2014). Combination of meal and exercise timing with a high‐fat diet influences energy expenditure and obesity in mice. Chronobiology International, 31, 959–975. 10.3109/07420528.2014.935785 25007387

[phy215044-bib-0027] Singh, R., Pattisapu, A., & Emery, M. S. (2020). US physical activity guidelines: Current state, impact and future directions. Trends in Cardiovascular Medicine, 30, 407–412. 10.1016/j.tcm.2019.10.002 31677904

[phy215044-bib-0028] Zachary, Z., Brianna, F., Brianna, L., Garrett, P., Jade, W., Alyssa, D., & Mikayla, K. (2020). Self‐quarantine and weight gain related risk factors during the COVID‐19 pandemic. Obesity Research & Clinical Practice, 14, 210–216. 10.1016/j.orcp.2020.05.004 32460966 PMC7241331

